# New data on the evolutionary history of the European bison (*Bison bonasus*) based on subfossil remains from Southeastern Europe

**DOI:** 10.1002/ece3.7241

**Published:** 2021-02-10

**Authors:** Boyko Neov, Nikolai Spassov, Latinka Hristova, Peter Hristov, Georgi Radoslavov

**Affiliations:** ^1^ Department of Animal Diversity and Resources Institute of Biodiversity and Ecosystem Research Bulgarian Academy of Sciences Sofia Bulgaria; ^2^ Palaeontology and Mineralogy Department National Museum of Natural History Bulgarian Academy of Sciences Sofia Bulgaria

**Keywords:** mitochondrial DNA, population structure, the Balkan Peninsula

## Abstract

The origin and evolutionary history of the European bison *Bison bonasus* (wisent) have become clearer after several morphological, genomic, and paleogenomic studies in the last few years, but these paleogenomic studies have raised new questions about the evolution of the species. Here, we present additional information about the population diversity of the species based on the analysis of new subfossil Holocene remains from the Balkan Peninsula. Seven ancient samples excavated from caves in Western Stara Planina in Bulgaria were investigated by mitochondrial D‐loop (HVR1) sequence analysis. The samples were dated to 3,800 years BP by radiocarbon analysis. Additionally, a phylogenetic analysis was performed to investigate the genetic relationship among the investigated samples and all mitochondrial DNA sequences from the genus Bison available in GenBank. The results clustered with the sequences from the extinct Holocene South‐Eastern (Balkan) wisent to the fossil Alpine population from France, Austria, and Switzerland, but not with those from the recent Central European (North Sea) one and the now extinct Caucasian population.

In conclusion, these data indicate that the Balkan wisent that existed in historical time represented a relict and probably an isolated population of the Late Pleistocene‐Holocene South‐Western mountainous population of the wisent.

## Graphical abstract

*Bison bonasus* remains from Ponor part of Western Stara Planina Mountain (Bulgaria). The data indicate that the Balkan wisent that existed in historical time represented a relict and probably isolated population of a distinct South European mountainous population of the wisent.

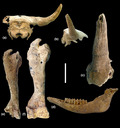

## 1 INTRODUCTION

The origin and evolution of the largest and one of the rarest extant European mammal species—the wisent (also called the European bison), *Bison bonasus* (Linnaeus, 1758)—has always been of interest to zoologists and paleontologists. Active research in this area dates back to the beginning of the last century (Flerov, 1979; Grange et al., 2018; Hilzheimer, 1918; Massilani et al., 2016; Palacio et al., 2017; Pucek, 1986; Soubrier et al., 2016; Spassov, 2016; Spassov & Stoytchev, 2003). The species had a wide historic geographic distribution throughout the European continent during the middle and late Holocene, ranging from France in the west to the Caucasus in the east, as demonstrated by studies using morphological methods, rock engravings analyses, and modern techniques for ancient DNA (aDNA). According to some conclusions, the European bison emerged for the first time after the Last Glacial Maximum (LGM, approximately 15,000 years ago) from a refuge in the South Caucasus and then spread into Central and West Europe at the onset of the Holocene (Massilani et al., 2016). This suggestion is based on circumstance since no genotypes of *Bison bonasus* have been found in ancient European samples before this period. However, other genetic and rock engraving studies suggest a much earlier time of arrival in Europe (11.7 kya) (Grange et al., 2018; Spassov & Stoytchev, 2003).

To date, two sublineages of the wisent have been recognized and called Bb1 and Bb2 (Grange et al., 2018). Bb1, also named Bison X, is believed to have belonged to the paleontological species *Bison schoetensacki* (Palacio et al., 2017), which corresponds to a lineage that went extinct at the onset of the Holocene. In contrast, Bb2 is the lineage appearing at the end of the Pleistocene that gave rise to recent wisent. It was established that the modern wisent Bb2 lineage was found in samples from periods and locations that differed from those where the Bb1 lineage was found (Massilani et al., 2016; Soubrier et al., 2016; Wecek et al., 2016).

The Bb2 lineage is rooted in two branches: the first one discovered in the specimens from France dating from the Early Holocene to the Middle Ages (Massilani et al., 2016), including the specimens from Austria (Wecek et al., 2016), and the second one encompassing all specimens from the Caucasus, the specimen from Switzerland and all present‐day wisents from Poland up to the year 1927 when wisents became extinct in the wild (Grange et al., 2018).

Information about the presence and distribution of the *B. bonasus* in historical times has been mainly based on ancient written sources (Benecke, 2005). The names used to describe the European bison have varied widely, which has created confusion and weakened the reliability of historical written sources (Benecke, 2005). For example, Aristotle used the word “bonasus” to describe the European bison in his reports on the history of animals (Aristotle & Balme, 1991). Aristotle described the animals as inhabiting the mountain regions of ancient Paeonia and Maedica (part of present‐day North Macedonia and Southwestern Bulgaria). In later years, between 1,450 and 1,850, “auerochs” was used as the official name of wisent/bison/zubr. There were attempts to eliminate this confusion in subsequent accounts by assigning the names “aerox” for aurochs and “bisont” for wisents (Ahrens, 1921). Yet, some twentieth century and medieval descriptions use the name “aurochs” for the European bison (Avebury, 1913; Von Lengerken, 1953). However, additional archaeological data are needed to resolve the extent of the European bison diversity and distribution in some uninvestigated regions, particularly in Europe. All these divergent historical references to wisents highlight that there was never a clear understanding of their exact origin and systematic position among animals.

To this day, data about the genetic structure of historical Balkan wisent population are lacking and the question about the origin of this population remains. This study aimed at contributing to the knowledge of the genetic structure and morphological data regarding the origin of the European wisent, based on sequence analysis of subfossil remains from Southeastern Europe (Bulgaria). The comparison of the results from this region to the other available bison genetic data fills an important gap in our understanding of the origin and migration processes of the European bison (wisent).

## 2 MATERIALS AND METHODS

### 2.1 Subfossil bone samples

**FIGURE 1:**
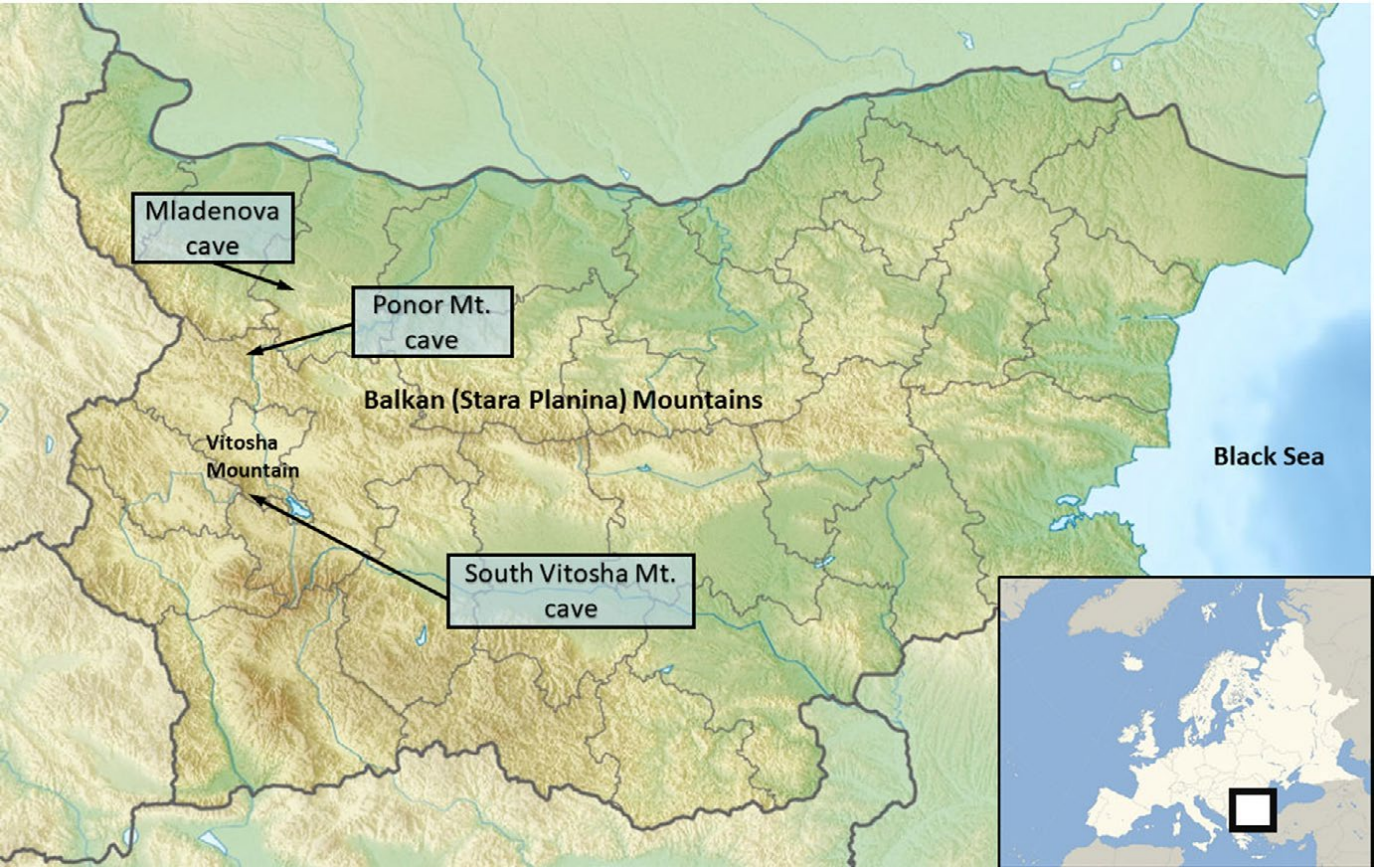
Locations of sample collection sites from the territory of Bulgaria. 5 bone samples were collected from Propastna and other caves in Ponor subrange of Stara Planina mts, 1 sample from Mladenova Dupka cave, and 1 sample from a cave in Vitosha Mountain

**FIGURE 2:**
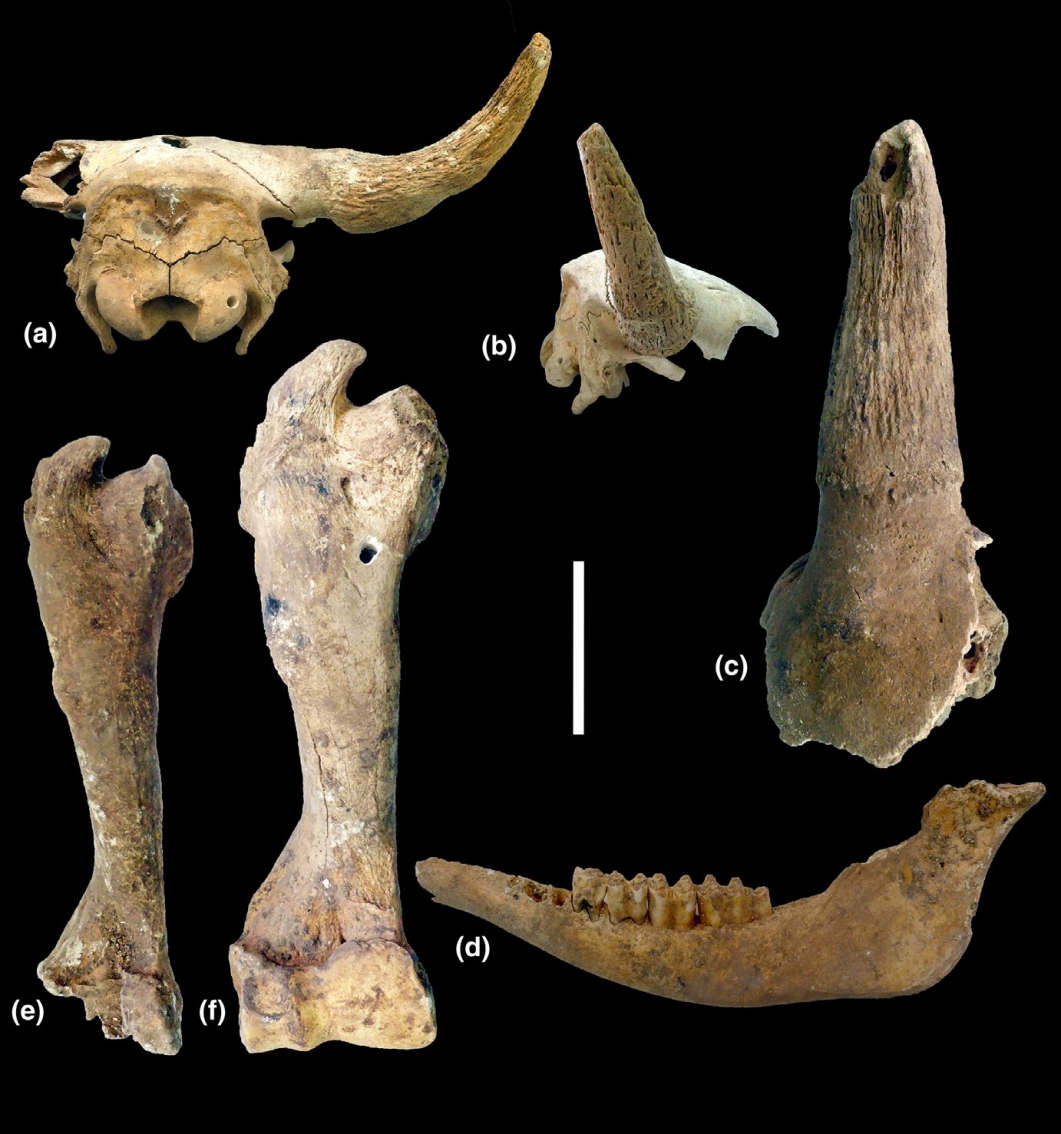
*Bison bonasus* remains from Ponor part of Western Stara Planina Mountain (Bulgaria), from the coll. of the National Museum of Natural History, Sofia (NMNHS). (a and b) Subadult male cranial fragment (SM3543), occipital and lateral views; (c) adult male cranial fragment (SM3542), frontal view; (d) left semimandible with p4‐m3 of a possibly female ind. in lateral view; (e) humerus of a female ind. in cranial view; (f) humerus of a male ind. in cranial view. Scale

Seven wisent samples from the collection of the National Museum of Natural History‐BAS (NMNHS) were used in the present study. The samples had been collected from three closely situated caves (Figures 1 and 2, Table S1). They cover the Late Pleistocene to the Middle/Late Holocene epochs. More information about the samples and descriptions is given in Table S1. Dating of the samples was based mainly on analysis of concomitant remains of other species. One bone sample (No. 7; Pon1) was sent to the Oxford Radiocarbon Accelerator Unit, Oxford University, for radiocarbon dating.

### 2.2 The wisent subfossil bone remains: locality and age

Seven wisent subfossil bone remains (adult male, adult female, and a young individual, probably male, with unfused cranial sutures) from three mountain locations were discovered in a vertical precipice cave in the region of the Ponor subrange of Stara Planina mts, which is a part of Western Stara Planina. At present, these remains are part of the collection of NMNHS, BAS. The subfossil bone remains include a small fragment of cranium with left horn core belonging to an adult male individual, a partial cranium with preserved occipital and frontal parts, and a right horn core belonging to a subadult individual, two humeri representing a male and a possibly female individual, and a left semimandible possibly belonging to the female individual (Figure 2, Table S4).

### 2.3 Description and comparison of the investigated bone material

The postcornual part of the skull (preserved in the subad. ind. coll. NMNHS‐SM3543) is less reduced in comparison with the genus *Bos* and stands out clearly behind the horn cores in the dorsal direction. The occipital surface is at a blunt (not sharp as in *Bos*) angle to the front surface. The frontal part is broad, slightly convex in occipital view. The horn cores are positioned relatively close to the orbits; they are rather short and their bases over the pedicles are broad and sharply narrowed at the tips. A strong longitudinal grove is seen on the intact dorsal surfaces of the adult male horn core (SM3542). The mandibular check teeth (SM3546) have rather smooth lingual surface. Their total length (female individual: alveolar L p4‐m3 = 161.3 mm) is larger than the size of a female specimen from the coll. of the NMNHS (L p2‐m3 = 148 mm). The distal articular surface of the humerus is strongly asymmetrical, with high medial part of the trochlea and low capitulum in cranial view, as well as with a sharp trochlear lateral rim. All these features are characteristic of *Bison bonasus* in comparison with the genus *Bos* (Godina et al., 1962; Spassov, 1992). The dimensions of the wisents (SM3544, ad. male and ad. Female SM3545) from the Ponor subrange are very large. The size of the horn core of the adult individual exceeds the regular size of recent male wisents (*B*. *bonasus*). Both the male and the presumed female humeri are also very large in size (Figure 2, Table S4, Figure S1) (Flerov, 1979; Pucek, 1986; Reshetov & Sukhanov, 1979; Sher, 1997; Brugal, 2016; Froese et al., 2017).

### 2.4 Radiocarbon dating of wisent bones

The methodology of the sample processing is explained in the works of Brock et al. (2010) and Ramsey et al. (2004). Since the concentration of Carbon‐14 in the atmosphere is not constant, and modern measurements of its half‐life are different from those originally estimated, all data obtained from a radiocarbon sample need to be calibrated. For calibration of the obtained results, we used the software Oxcal (v4.2.4 и v. 4.3.2) designed by Ramsey and Lee (2013), and for the calibration curve used to perform the calibrations—the software IntCal 13 (Reimer et al., 2013).

### 2.5 Sample preparation and ancient DNA isolation

The genetic material was isolated according to the protocol of Yang et al. (1998) with modifications (Dzhebir et al., 2018; Hristov et al., 2017). Briefly, about 0.5 g of bone samples powder was treated with 5 ml lysis buffer for 48–36 hr at 56°C. Ancient DNA was isolated from a digested solution by silicone membrane columns (GeneMatrix, E3520, EURx, Poland), eluted in 100 μl TE buffer and stored at −20°C before use. More information about aDNA isolation is given in Supplementary material S1.

### 2.6 PCR amplification and sequencing

We used PCR amplification of two overlapping fragments of the informative HVRI region on the mitochondrial D‐loop region. To achieve best results, aDNA amplification was performed in two independent steps, using the nested PCR method with specific primers (Table S2) (Massilani et al., 2016). The first nested PCR primer sets covered the 15,765 bp–16,024 bp (259 bp) and the 15,884 bp–16,274 bp (390 bp) regions of the HVRI while the second nested PCR primer sets amplified internal and more specific short DNA products with sizes between 140 bp and 180 bp (Table S2). For negative control, we used a single‐step PCR assay with primer sets from the first nested PCR with and without template DNA to confirm the lack of contamination of the samples with exogenous DNA. The position of the primers was relative to the wisent reference sequence NC_014044 (Zeyland et al., 2012).

Additionally, shorter fragments were amplified following the methodology described by Massilani et al. (2016) in order to verify the consistency of the results.

The PCR products were visualized on a prestained (SimplySafe™, EURx Ltd., Poland) 1% agarose gel electrophoresis under UV light. The successfully amplified products were purified by a PCR purification kit (Gene Matrix, PCR clean‐up kit, EURx, Poland) and sequenced by a PlateSeq kit (Eurofins Genomics Ebersberg, Germany).

### 2.7 Phylogenetic reconstruction

The obtained sequences were manually edited and aligned by MEGA software version 7.0 (Kumar et al., 2016), using the wisent mtDNA sequence NC_014044 (Zeyland et al., 2012) as a reference. Sequences were analyzed by polymorphic SNPs. The phylogenetic analysis was based on the subfossil wisent bone samples used in this study as well as on all available in GenBank ancient and modern DNA Bison species sequences (Table S3) (Grange et al., 2018; Massilani et al., 2016; Soubrier et al., 2016; Wecek et al., 2016). Ancient and recent mtDNA sequences were characterized using network analysis—NETWORK 4.5.1.6 (Fluxus Technology Ltd., Suffolk, England) (available at http://fluxusengineering.com).

The sequences obtained in this study were deposited in the National Center for Biotechnology Information (NCBI) GenBank database under accession numbers NCBI: MG808411–MG808413.

## 3 RESULTS

### 3.1 Age determination of wisent samples

**FIGURE 3:**
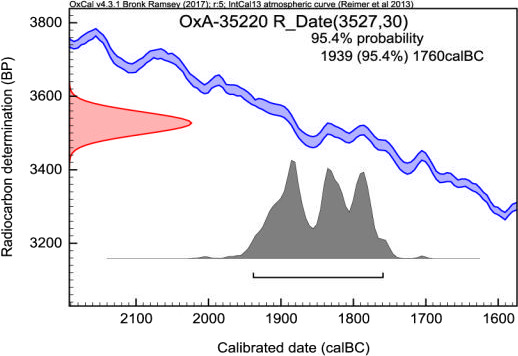
The radiocarbon calibrated dates for sample OxA‐35220; No.7 of wisent bone from Ponor part of Western Stara Planina Mountain, obtained in the Oxford laboratory (Oxford Radiocarbon Accelerator Unit). The red curve on the left represents the measured sample signal (in years before present), the blue curve represents the calibration curve, and the resulting probability distribution is on the bottom, suggesting a 95% chance of sample originating from 1,940 to 1,760 years BC. The gray histogram shows possible ages for the sample (the higher the histogram, the more likely that age is)

The age of one bone sample (No. 7; Pon1) was determined by radiocarbon dating (OxA‐35220). The bone fragment showed an age of 3,527 + 30 years, before calibration, and an age of 3,890–3,710 years, after calibration (Figure 3).

### 3.2 Ancient DNA analysis

**FIGURE 4:**
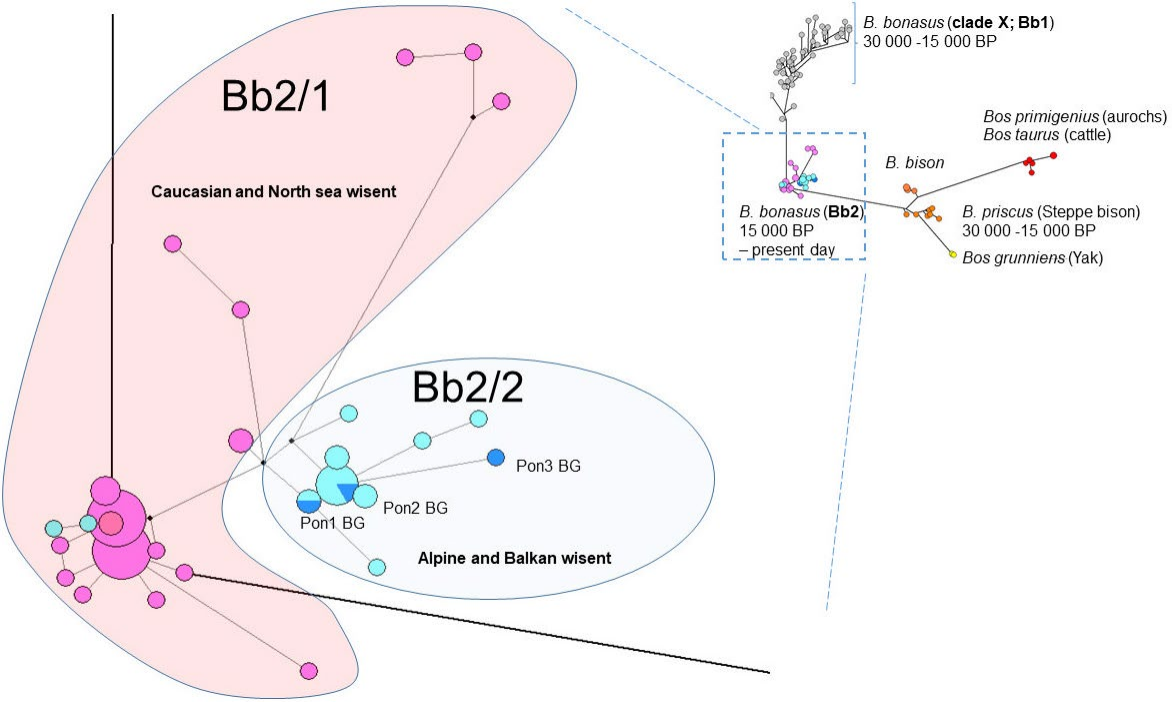
The median‐joining network of the mtDNA haplotypes from *Bos* and *Bison*. The clade Bb2 is presented with two lineages: Pink area assigned to Caucasian and North Sea wisent, while blue circle includes Alpine (light blue) and the Balkan (dark blue) wisent. Circle areas are proportional to haplotype frequencies

Ancient DNA was successfully amplified from 3 of the 7 samples tested. All successfully amplified samples were from the Early Holocene (Stara Planina mts, Ponor subrange) (Figures 1 and 2, Table S1). After proper processing, the resulting fragments were estimated to 394–513 bp in size, part of the beginning of D‐loop region. The phylogenetic analysis showed the highest homology of the studied sequences with wisents from France (Massilani et al., 2016), Austria (Wecek et al., 2016), and Switzerland (Soubrier et al., 2016) (Figure 4, Table S5). All these wisents belong to the specific Alpine Region *Bison bonasus* assigned by us as Bb2/2 lineage in contrast to the typical for the Caucasian and North Sea regions lineage named Bb2/1 (Figure 4).

## 4 DISCUSSION

### 4.1 European bison population during the late Pleistocene and the Holocene

According to current understanding, the European bison (*Bison bonasus*), or wisent, can be subdivided into two genetically distinct lineages: Late Pleistocene (30,000–15,000 BP) (Bb1; clade X) and Late Pleistocene to present day (Bb2 clade) (Grange et al., 2018; Massilani et al., 2016; Soubrier et al., 2016; Wecek et al., 2016). The wisent is morphologically and genetically distinct from coinhabiting species: steppe bison (*Bison priscus*) and aurochs (*Bos primigenius*) and its domesticated form *Bos taurus* (Figure 4).

Specific for Bb2 lineages is that they include two genetically and geographically distinct wisent groups (branches). The first one, named Bb2/1, which is found in the North Sea and the Caucasian region, includes wisents inhabiting today mainly plain forests in Russia, Poland, and Georgia (Grange et al., 2018; Massilani et al., 2016; Soubrier et al., 2016; Wecek et al., 2016). It is important from a taxonomic viewpoint to note that the described Holocene Central European wisent *B. bonasus hungarorum* Kretzoi (Flerov, 1979) is closer to the steppe form from the forests of Poland and represents not more than an isolate of this lineage.

The second Bb2/2 lineage is related to Alpine parts of Europe and is known only from ancient specimens (France, Austria, and Switzerland) belongs to the time span from the Pleistocene/Holocene boundary till the Middle Ages (Grange et al., 2018; Massilani et al., 2016; Soubrier et al., 2016; Wecek et al., 2016).

Our results (the first from the Balkans) have revealed the closest genetic relationships with the Central and Western (Alpine) European wisent. Given that wisents from Bialowieza and from Caucasus (which have distinct morphological and ecological features and which are considered different subspecies: Flerov, 1979; Pucek, 1986) belong to one and the same genetic branch, we have reason to assume that the Alpine and Balkan European branch should be a separate, extinct subspecies of the wisent. In this case, the separation of this branch must have taken place relatively long before the end of the Pleistocene. The presumed route of dispersal of the group is from the Caucasus and Asia Minor through the Balkans to the western territories of Europe.

## 5 CONCLUSIONS

In conclusion, our findings have expanded the limited existing data related to the geographical area of the noted Alpine population of the wisent to the east till the Balkan region. Our data indicate that the Balkan wisent that existed in historical time represented a relict and probably isolated population which is a distinct European mountainous population of the wisent. These results are not contradictory to the hypothesis that *Bison bonasus* had originated somewhere in the area between South (South‐Eastern) Europe and the Middle East and Caucasus much before the end of the Pleistocene (Spassov, 2016). To the present day, this area remains insufficiently studied from the viewpoint of Pleistocene—Holocene bison history.

## CONFLICT OF INTEREST

The authors have no competing interests to declare.

## AUTHOR CONTRIBUTIONS

**Boyko Neov:** Formal analysis (supporting); investigation (supporting); writing‐review & editing (equal). **Latinka Hristova:** Formal analysis (supporting); investigation (supporting); visualization (equal); writing‐original draft (supporting); writing‐review & editing (supporting). **Nikolai Spassov:** Conceptualization (equal); funding acquisition (equal); resources (equal); supervision (lead); validation (equal); writing‐original draft (equal); writing‐review & editing (equal). **Peter Hristov:** Conceptualization (equal); data curation (equal); formal analysis (equal); investigation (equal); software (equal); writing‐original draft (equal); writing‐review & editing (equal). **Georgi Radoslavov:** Conceptualization (equal); formal analysis (equal); funding acquisition (lead); investigation (equal); resources (equal); software (equal); supervision (equal); visualization (equal); writing‐original draft (lead); writing‐review & editing (lead).

## Supporting information

Fig S1

Table S1

Table S2

Table S3

Table S4

Table S5

Supplementary Material

## Data Availability

All relevant data are available within the manuscript Supporting Information files, and sequences are deposited on the GenBank public repository under the accession numbers MG808411–MG808413.
