# Supplementary material for: New data on the evolutionary history of the European bison (Bison bonasus) based on subfossil remains from Southeastern Europe
Source: Ecol Evol. 2021 Feb 10;11(6):2842–8. doi: 10.1002/ece3.7241 (PMC7981210; doi:10.1002/ece3.7241)
Supplement: Supplementary file 1 — Fig S1 [file ECE3-11-2842-s006.docx]

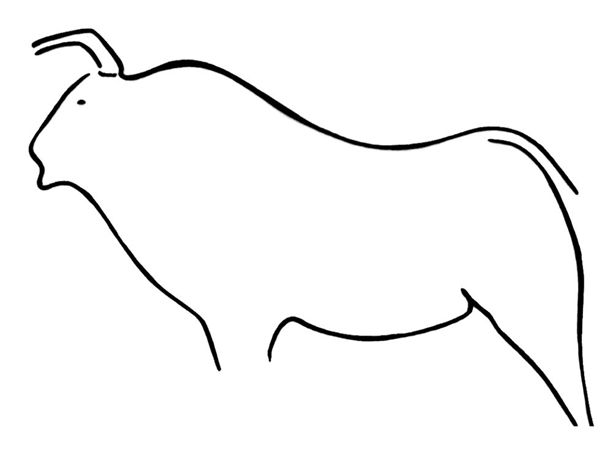


**Supplementary Figure S1.** Wisent (*Bison bonasus*) rock engraving: after Spassov & Stoychev, 2003 (Pair-non-Pair Cave, Gironde, France. Early Aurignacian (more than 37 000 years BP),
