# Supplementary material for: New data on the evolutionary history of the European bison (Bison bonasus) based on subfossil remains from Southeastern Europe
Source: Ecol Evol. 2021 Feb 10;11(6):2842–8. doi: 10.1002/ece3.7241 (PMC7981210; doi:10.1002/ece3.7241)
Supplement: Supplementary file 2 — Table S1 [file ECE3-11-2842-s003.docx]

**Supplementary Table S1.** List of all Bulgarian wisent samples used in ancient DNA analysis.

| **No** | **Periodization** | **Collection number** | **Source** | **Abbreviation** | **Skeletal element** | **Product size, bp** | **GenBank Acc. Number** |
| --- | --- | --- | --- | --- | --- | --- | --- |
| 1 | Early Holocene  (3527  BP) | SM3543 | Stara Planina mts. Ponor subrange | Pon1 | cranium + cornu | 394 | MG808411 |
| 2 |  | SM3542 | Stara Planina mts. Ponor subrange | Pon2 | cranium + cornu | 513 | MG808412 |
| 3 |  | SM3546 | Stara Planina mts. Ponor subrange | Pon3 | mandibula | 513 | MG808413 |
| 4 | Early Holocene | SM3544 | Stara Planina mts. Ponor subrange | - | humerus | - | - |
| 5 | Late Pleistocene |  | Stara Planina mts., Propastna cave, Ponor subrange | - | metacarpus | - | - |
| 6 | Early Holocene /Late Pleistocene |  | Stara Planina mts, Mladenova cave | - | cornu | - | - |
| 7 | Late Pleistocene | FM1985 | South Vitosha Mountain | - | metacarpus | - | - |
