# Supplementary material for: New data on the evolutionary history of the European bison (Bison bonasus) based on subfossil remains from Southeastern Europe
Source: Ecol Evol. 2021 Feb 10;11(6):2842–8. doi: 10.1002/ece3.7241 (PMC7981210; doi:10.1002/ece3.7241)
Supplement: Supplementary file 3 — Table S2 [file ECE3-11-2842-s004.docx]

**Supplementary Table S3.** Primers used for amplification of the D-loop region of mtDNA. The position of the primers is relative to the reference sequence NC_014044 (Zeyland et al., 2012). * Primer used for Nested PCR and sequencing

| **Region** | **Primer**  **sense/antisense** | **Sequence**  **5'-3'** | **Reference** | **Product size, bp** |
| --- | --- | --- | --- | --- |
| I. | Bb_Bon2_S_15765 | tgaagttctatttaaactattccctg | Massilani et al., 2016 | 259 |
|  | Bb_Bon1_As_16024 | gcatggggcatataatttaatgtact |  |  |
| I.1. | Bb_Bon1_S_15884* | aaatattacaaacaccactagctaac |  | 140 |
|  | Bb_Bon1_As_16024* | gcatggggcatataatttaatgtact |  |  |
| I.2. | Bb_Bon2_S_15765* | tgaagttctatttaaactattccctg |  | 140 |
|  | Bb_Bon2_as_15905* | gctagtggtgtttgtaatatttggt |  |  |
| II. | Bb_Bon1_S_15884 | aaatattacaaacaccactagctaac |  | 390 |
|  | Bb_Bon3_as_16274 | gccctgaagaaagaaccagat |  |  |
| II.1. | Bb_BB3r4m_S -16015* | tgccccatgcatataagcaag |  | 151 |
|  | Bb_BB3r4m_As-16166* | tcacgcggcatggtayttaag |  |  |
| II.2. | Bb_Bon1_S_15884* | aaatattacaaacaccactagctaac |  | 182 |
|  | Bb_BB3r4m_As-16166* | tcacgcggcatggtayttaag |  |  |
| II.3. | Bb_BB3r4m_S -16015* | tgccccatgcatataagcaag |  | 259 |
|  | Bb_Bon3_as_16274* | gccctgaagaaagaaccagat |  |  |

**References**

Massilani, D., Guimaraes, S., Brugal, J. P., Bennett, E. A., Tokarska, M., Arbogast, R. M., Baryshnikov, G., Boeskorov, G., Castel, J.-C., Davydov, S., Madelaine, S., Putelat, O., Spasskaya, N. N., Uerpmann, H.-P., Grange, T., & Geig, E.-M. (2016). Past climate changes, population dynamics and the origin of Bison in Europe. *BMC Biology*, **14**, 93

Zeyland, J., Wolko, L., Lipinski, D., Wozniak, A., Nowak, A., Szalata, M., Bocianowski, J., & Slomski, R. (2012) Tracking of wisent-bison-yak mitochondrial evolution. *Journal of Applied Genetics,* **53**, 317-322.
