# Supplementary material for: New data on the evolutionary history of the European bison (Bison bonasus) based on subfossil remains from Southeastern Europe
Source: Ecol Evol. 2021 Feb 10;11(6):2842–8. doi: 10.1002/ece3.7241 (PMC7981210; doi:10.1002/ece3.7241)
Supplement: Supplementary file 5 — Table S4 [file ECE3-11-2842-s002.docx]

**Supplementary Table S4**. Comparative dimensions of the horn cores and humeri of the wisents from Ponor mountain, Bulgaria (comparative data from Flerov, 1979; Reshetov & Sukhanov, 1979; Pucek, 1986; Brugal, 2016). All measurements are in mm.

| Taxa  Features | *B*. *bonasus bonasus* | | | | *B*. *bonasus caucasicus* | | *B*. *bonasus*  from Ponor | |
| --- | --- | --- | --- | --- | --- | --- | --- | --- |
|  | males | | females | | males | females | male | Female and subad. |
|  | variation | mean | variation | mean | variation | variation |  |  |
| Max. length of humerus | 327-370  (n=26) | 349 | 301-318 | 314 | 325 | 300 | 379 | 344 female |
| Circumference of the horn core | 195-250 |  | 130-168 |  |  |  | 275 |  |
| Antero-post. Diameter of horn core |  | 65-80  (n=5) |  |  |  |  | 90 | 62 subad. male |
| Dorso-ventral D. of horn core |  | 70-88  (n=5) |  |  |  |  | 94 | 68  subad. male |

**References**

Flerov, K. K. (1979). Sistematika i evolyutsiya. In Zubr: Morfologiya, sistematika, evolyutsiya, ekologiya. Moscow: Nauka, pp. 9 – 127.

Pucek, Z. (1986). *Bison bonasus* – Wisent. In: Niethammer J. & Krapp F. (eds.). Handbuch der Saugetiere Europas. Aula Verlag GmbH, Wiesbaden. Band 2/II (Paarhufer), pp. 278-315.

Reshetov, V., & Sukhanov, V. (1979). Postcranial skeleton. In European Bison—Morphology, Systematics, Evolution, Ecology (in Russian); Sokolov, V.E., Ed.; Nauka (USSR Ac. of Sc.): Moscou, Russia, 1979; pp. 142 –195 (in Russian).

Brugal, J. P. (2016). Paleohistory of an Aurignacian bison skull from Régismont-le-Haut (Hérault): from nature to value. *PALEO. Revue d'archéologie préhistorique*, **27**, 62-82.
