# Supplementary material for: New data on the evolutionary history of the European bison (Bison bonasus) based on subfossil remains from Southeastern Europe
Source: Ecol Evol. 2021 Feb 10;11(6):2842–8. doi: 10.1002/ece3.7241 (PMC7981210; doi:10.1002/ece3.7241)
Supplement: Supplementary file 6 — Table S5 [file ECE3-11-2842-s007.docx]

**Supplementary Table S5**. Highly homology sequences of Alpian group *Bison bonasus* (Bb2).

| **GenBank Acc Number** | **Coll number** | **Country** | **years old** | **Reference** |
| --- | --- | --- | --- | --- |
| KX898009 | GRAL125 | France: Lot, Igue du Gral  France: Lot, Igue du Gral | 12400-11800 | Massilani et al., 2016 |
| KX898008 | GRAL76 |  | 12100-11700 |  |
| KX870179 | GRAL76  GRAL01-P47d4-76 |  | 12100-11700 |  |
| KX870178 | GRAL125  P47-dec006-125 |  | 12400-11800 |  |
| KX870175 | Ost487 | France: Alsace, Ostheim Birgelsgaerten | 1400-1300 |  |
| KX870174 | Ost486 | France: Alsace, Ostheim Birgelsgaerten | 1400-1300 |  |
| KX870177 | CHL16 | France: Jura, Chalain | 5200 |  |
| KX870176 | CHL8 | France: Jura, Chalain | 5200 |  |
| LT599639 | BS600 | Austria:Steiermark | 3577-3831 | Soubrier et al., 2016 |
| LT599638 | BS593 | Austria:Steiermark | 5707-5940 |  |
| LT599644 | A15665 | Switzerland:La Brevine | 3843-3990 |  |
| LT599640 | BS607 | Austria:Oberoesterreich | 1227-1369 |  |
| KX553931 | Bb2/BS589 | Styria, Austria | 1987–1886 | Wecek et al., 2016 |
| KX553932 | Bb3/BS607 | Upper Austria, Austria | 1338-1265 |  |
| KX553930 | Bb1/BS587 | Styria, Austria | 1511–1302 |  |

**References**

Massilani, D., Guimaraes, S., Brugal, J. P., Bennett, E. A., Tokarska, M., Arbogast, R. M., Baryshnikov, G., Boeskorov, G., Castel, J.-C., Davydov, S., Madelaine, S., Putelat, O., Spasskaya, N. N., Uerpmann, H.-P., Grange, T., & Geig, E.-M. (2016). Past climate changes, population dynamics and the origin of Bison in Europe. *BMC Biology*, **14**, 93.

Soubrier, J., Gower, G., Chen, K., Richards, S. M., Llamas, B., Mitchell, K. J., & Bollongino, R. (2016). Early cave art and ancient DNA record the origin of European bison. *Nature Communications*, 7, 13158.

Wecek, K., Hartmann, S., Paijmans, J. L., Taron, U., Xenikoudakis, G., Cahill, J. A., Heintzman, P. D., Shapiro, B., Baryshnikov, G., Bunevich, A. N., Crees, J. J., Dobosz, R., Manaserian, N., Okarma, H., Tokarska, M., Turvey, S. T., Wójcik, J. M., Żyła, W., Szymura, J. M., Hofreiter, M., & Barlow, A. (2016). Complex admixture preceded and followed the extinction of wisent in the wild. *Molecular Biology and Evolution*, **34**, 598-612.
