# Supplementary material for: New data on the evolutionary history of the European bison (Bison bonasus) based on subfossil remains from Southeastern Europe
Source: Ecol Evol. 2021 Feb 10;11(6):2842–8. doi: 10.1002/ece3.7241 (PMC7981210; doi:10.1002/ece3.7241)
Supplement: Supplementary file 7 — Supplementary Material [file ECE3-11-2842-s005.docx]

**Protocols for working with ancient DNA**

**Ancient DNA isolation**

Due to the specific features of working with ancient DNA in the laboratories of the Institute of Biodiversity and Ecosystem Research (IBER-BAS) certain conditions were created to prevent contamination with exogenous DNA and to ensure the authenticity of the results. All experiments were performed according to standard precautions in specialized and territorially distinct laboratories for aDNA work: bone material processing, DNA isolation and PCR amplification (Paabo et al., 2004; Willerslev & Cooper, 2005). Briefly, this included: establishing independent laboratories (premises and buildings) for working with ancient DNA, treating surfaces and solutions with UV radiation (45 W, 72 h), heat treatment (over 180 ºC, 12 h), acid treatment (2.5M HCl, 48 h) and/or sodium hypochlorite (40%, 48 h), washing with ultrapure water and air filtration in the premises. The disposable plastic consumables and disposable safety devices and solutions are licensed to work with human DNA.

The genetic material was isolated according to the protocol of Yang et al. (1998) with minor modifications (Hristov et al., 2017; Dzhebir et al., 2018). To prevent contamination of the bone surface by foreign DNA, the samples were treated sequentially with sodium hypochlorite (40%), 2% hydrochloric acid, and washed with ultrapure H_2_O several times. After drying for 24-48 hours in UV-irradiation medium and constant air filtration, the surface layer was removed, and then bone powder was obtained by Dremel (Grinding A 11 basic analytical mill, Germany), which was further homogenized in metal mortars. Treatment of bone material for isolation of the aDNA was performed from 400-500 mg of bone powder dissolved in 5 ml of lysis buffer (0.5M EDTA, 2% Sodium dodecyl sulfate, 0.1M Tris pH8, 10 μl/ml Mercaptoethanol, 20 μl/ml Proteinase K). The samples were incubated in a hybridizer (Hybridiser HB-2D, Techne, UK) at constant rotation at 56°C for 36-48 hours. The samples were centrifuged at 5000 rpm for one hour, after which the supernatant was filtered through 0.45μm filters and transferred to 50 ml tubes. DNA isolation was performed by silicone membrane technology, including the use of DNA isolation columns (GeneMatrix, E3520, EURx, Poland) and 5M GuSCN (Sigma-Aldrich) binding reagent V/V and 20% EtOH. The aDNA bound to the silica columns was purified twice with a 70% ethanol wash solution and dissolved in ultrapure water. The isolated aDNA was stored at -20°C.

**PCR amplification and sequencing**

We used a nested PCR reaction to amplify 2 overlapping fragments of the HVRI region. The PCR products of the first nested PCR primer sets were: 15765 bp – 16024 bp (259 bp) and 15884 bp – 16274 bp (390 bp). The second PCR primer sets amplified the fragments that were used for the sequencing of products with sizes between 140 bp and 180 bp (Supplementary Table S3). The position of the primers is relative to the wisent reference sequence NC_014044 (Zeyland et al., 2012). For negative control we used primer sets from the first nested PCR reaction with and without template DNA.

All PCR reactions were performed with 10ng/μl DNA in a final volume of 50 μl by using NZYTaq Colourless Master Mix (Cat No – MB040, NZYTech, Portugal).

PCR reactions were performed under the following conditions: initial denaturation at 94°C for 5 min.; 40 cycles of denaturation at 94°C for 30 seconds, primers hybridization – 50°C for 30 seconds, elongation at 72°C for 1 minute; and final elongation at 72°C for 10 min. The amplified fragments were separated and visualized on 2% agarose gel electrophoresis.

**References**

Dzhebir, G., Yordanov, G., Yankova, I., Sirakova, D., Petrova, M., Neov, B., Hristov, P., Radoslavov, G., Hristova, L., & Spassov, N. (2018). Comparative genetic analysis of subfossil wild horses (from the Neolithic Age and Early Bronze Age) and present-day domestic horses from Bulgaria. *Historia naturalis bulgarica*, **25**, 3-10.

Hristov, P., Spassov, N., Iliev, N., & Radoslavov, G. (2017). An independent event of Neolithic cattle domestication on the South-eastern Balkans: evidence from prehistoric aurochs and cattle populations. *Mitochondrial DNA Part A: DNA Mapping, Sequencing, and Analysis* **8**, 383-39.

Pääbo, S., Poinar, H., Serre, D., Jaenicke-Després, V., Hebler, J., Rohland, N., & Hofreiter, M. (2004). Genetic analyses from ancient DNA. *Annual Review of Genetics*, **38**, 645-679.

Willerslev, E., & Cooper, A. (2005). Ancient DNA. *Proceedings of the Royal Society B: Biological Sciences*, **272**, 3-16.

Yang, D. Y., Eng, B., Waye, J. S., Dudar, J. C., & Saunders, S. R. (1998). Improved DNA extraction from ancient bones using silica‐based spin columns. *American* *Journal of Physical Anthropology*, **105**, 539-543.

Zeyland, J., Wolko, L., Lipinski, D., Wozniak, A., Nowak, A., Szalata, M., & Slomski,R. (2012) Tracking of wisent-bison-yak mitochondrial evolution. *Journal of Applied Genetics,* **53**, 317-322.
